# Organic nitrogen rearranges both structure and activity of the soil-borne microbial seedbank

**DOI:** 10.1038/srep42634

**Published:** 2017-02-15

**Authors:** Márcio F. A. Leite, Yao Pan, Jaap Bloem, Hein ten Berge, Eiko E. Kuramae

**Affiliations:** 1 Netherlands Institute of Ecology (NIOO/KNAW), Department of Microbial Ecology, Wageningen, The Netherlands; 2 Agroecology Program of Maranhão State University – UEMA, São Luís – MA, Brazil; 3 Alterra, Wageningen UR, The Netherlands; 4 Plant Research International, Wageningen UR, The Netherlands.

## Abstract

Use of organic amendments is a valuable strategy for crop production. However, it remains unclear how organic amendments shape both soil microbial community structure and activity, and how these changes impact nutrient mineralization rates. We evaluated the effect of various organic amendments, which range in Carbon/Nitrogen (C/N) ratio and degradability, on the soil microbiome in a mesocosm study at 32, 69 and 132 days. Soil samples were collected to determine community structure (assessed by 16S and 18S rRNA gene sequences), microbial biomass (fungi and bacteria), microbial activity (leucine incorporation and active hyphal length), and carbon and nitrogen mineralization rates. We considered the microbial soil DNA as the microbial seedbank. High C/N ratio favored fungal presence, while low C/N favored dominance of bacterial populations. Our results suggest that organic amendments shape the soil microbial community structure through a feedback mechanism by which microbial activity responds to changing organic inputs and rearranges composition of the microbial seedbank. We hypothesize that the microbial seedbank composition responds to changing organic inputs according to the resistance and resilience of individual species, while changes in microbial activity may result in increases or decreases in availability of various soil nutrients that affect plant nutrient uptake.

The application of organic amendments, as an alternative or complement to mineral fertilization, is intended to increase soil fertility^1^, and especially nitrogen level, which is a yield-limiting factor. However, when compared to mineral fertilizers, use of organic amendments presents a different challenge in that it depends on the capacity of soil microbes to decompose the organic input to release nutrients for plant uptake^2^. In contrast, mineral fertilization provides readily available nutrients for plant growth^3^.

Organic amendment decomposition depends on the content of polymeric compounds^4^, which determine the quality of the organic material^5^. This quality, primarily defined by the C/N ratio and degradability, affects the composition and activity of the soil microbial community. Organic material with a C/N ratio less than 25/1 leads to mineralization due to the excess of N relative to available C and, consequently, N is primarily released as NH_4_^+ ^6. However, a C/N ratio greater than 25/1 tends to immobilize mineral N^7^ because microbial biomass tends to retain N or extract it from the inorganic pool^6^. The decomposition process relies on the degradation of the labile fraction and concentration of molecules resistant to biodegradation according to the fiber content (cellulose, hemicellulose, lignin, etc.)^8^. Most of the *Ascomycota* and *Basidiomycota* fungi, as well as several bacterial species in both filamentous (*e.g., Streptomyces, Micromonospora*) and non-filamentous (*e.g., Bacillus, Cellulomonas*) genera^9^, possess the capability to degrade cellulose^10^. As a consequence, some soil microbial populations might face resource competition during decomposition that can change the community structure according to changing available resources^11^.

Recent studies have contributed to our understanding of organic decomposition by describing soil organic matter formation and chemical composition of stable C and N forms^12^ and have highlighted the roles of both labile and recalcitrant plant compounds^13^. Results from these studies have led to the establishment of a framework that integrates the processes of plant organic matter decomposition and soil organic matter stabilization^2^. However, there is a lack of information about how the quality of organic material affects changes in the microbial community (fungi and bacteria), especially considering that each community has its own capacity for resistance and resilience^14^. Here we define microbial community resilience as the capacity of the microbial community to recover to its original structure after a perturbation, while resistance is defined as the ability of a microbial community to endure the changes after a perturbation. The community stability of soil microorganisms is even more challenging to study given that not all microbes in the soil microbial ‘seedbank’ are active^15^ with respect to biodegradation. Accordingly, in order to understand the influences of organic amendments on the soil microbial community, researches need to take into account how changes in the active microbial community further influence the overall composition of the microbial community itself in terms of resilience and resistance. In this context, Shade, *et al*.^14^ provided key concepts and suggested that meta-omics approaches could achieve a better description of these phenomena.

Moreover, nutrient inputs shift the microbial community based on not only taxonomy, but also on functional traits^16^ and can favour copiotrophic or oligotrophic microorganisms, depending on the inputs added^17^. Organic amendments can shift the composition of the active microbial community and, consequently, that of the whole soil microbial community, but we still lack detailed information about how this process occurs. Thus, monitoring changes in both the soil microbial community structure and activity of the soil microbial community after application of organic fertilizers is essential for understanding not only the soil nutrient availability, especially nitrogen^18^, but also for understanding how the organic amendment affects community stability. Community shifts may also follow the response of microbial biomass and activity due to organic amendments and mineral fertilizers, thus altering the total soil community and ultimately affecting nutrient uptake. However, to date, no studies have investigated such microbial shifts caused by organic amendments.

We hypothesize that organic amendments induce qualitative changes to the microbial soil community and that this shift in community is stronger with respect to the active community component than for the entire microbial seedbank. In order to test this hypothesis, we conducted a mesocosm study using various organic amendments that differed in both C/N ratio and degradability and assessed the resulting changes in both bacterial and fungal communities. Finally, we discuss how changes in microbial community activity can interfere with community stability of the soil microbial seedbank.

## Results

### Organic amendment effect on microbial community structure and activity

We established that organic fertilization treatments alter the composition of both the soil bacterial and fungal communities. The Between-Class Analyses (BCAs) revealed the amount of total data variability relative to the adopted grouping criteria. The joint group effects of treatment (N source) and time (32, 69, 132 days) corresponded to 56.03% and 44.25% (*p* = 0.001) of the total inertia from bacterial and fungal community structures, respectively (Supplementary Fig. S1a,b). However, when we evaluated these data sets independently by treatment and time, the grouping factors reduced the total retained data variability. Nitrogen source and time retained accounted for 21.82% (*p* = 0.003) and 9.38% (*p* = 0.001) of the total variance, respectively, for bacterial community structure (Supplementary Fig. S2a,b). Both N source and time also contributed less to the total data variability for the fungal community (23.97% and 5.37%, respectively) (Supplementary Fig. S3a,b) with significant difference from the null model for both analyses (*p* = 0.002 and *p* = 0.002).

According to the STATICO analysis, both bacterial and fungal communities varied similarly with respect to time (Supplementary Fig. S4) and interacted significantly with each other only for the first time point at 32 d (*p* = 0.002). The interstructure (Supplementary Fig. S4a) showed that the first dimension projected most of the time co-inertia. The same pattern was observed for COSTATIS analysis, which revealed a group mixing between the control (Control and Dcontrol) and the remaining treatments (Supplementary Fig. S5), indicating the existence of a co-structure between bacterial and fungal communities. However, despite the high covariance (RV = 96.17%), this relation is likely random (*p* = 0.4024). Thus, we found a significant effect of time for both bacterial and fungal communities, but these two communities did not covary over time.

**Figure 1:**
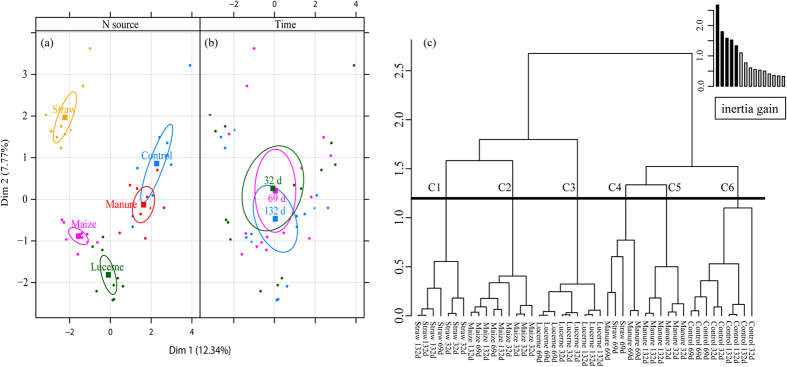
Multiple factor analysis of the bacterial and fungal communities (Hellinger-transformed data) together with the microbial biomass and microbial activity, according to the (**a**) organic amendment (Control, Manure, Lucerne, Corn and Straw) and (**b**) time (32, 69 and 132 days) with (**c**) Hierarchy of Multiple Factor Analysis considering the clusters (C1, C2, C3, C4, C5, C6) of corresponding variables and the amount of inertia gained in the clustering cut-off.

Figure 1 illustrates the global PCA from microbial community structure (bacteria and fungi) together with the variables of microbial biomass and microbial activity (bacterial leucine incorporation and active fungal hyphae). Inclusion of the microbial biomass and activity data contributed to improved distinction between the treatments. The Manure treatment was positioned close to the Control, while Lucerne and Maize microbial communities grouped together and the Straw microbial community differed from the others (Fig. 1a). In summary, time had a smaller effect when compared to the treatments and contributed less to explain the differences between the microbial communities as shown by BCA, STATICO and COSTATIS analyses.

The microbial community of Maize, Lucerne and Straw treatments positioned together, while the other treatments (Manure, Control and DControl) formed a separated group (Supplementary Fig. S6a,b). The microbial communities measured at day 132 were different from those at days 32 and 69. The Hierarchical Clustering of a Multiple Factor Analysis (HMFA) revealed four different clusters: cluster 1, representing the treatments with Manure; cluster 2, jointed DControl and Control; cluster 3, the Straw treatment; cluster 4, both Lucerne and Maize treatments (Supplementary Fig. S6c). This result points out that the changes in microbial community due to mineral fertilizer amendment are apparently the same at both doses.

**Figure 2:**
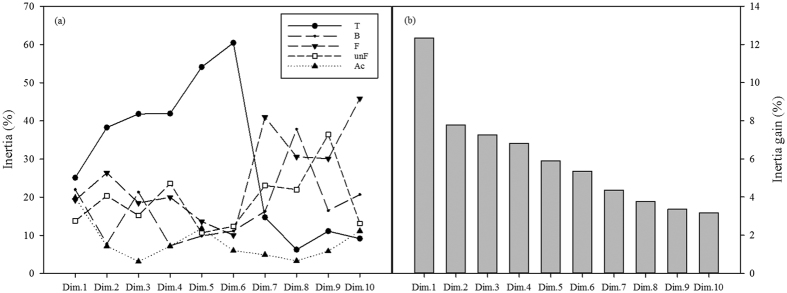
Percentage of inertia for each of the first 10 dimensions from the Multiple factor analysis of the bacterial and fungal communities (Hellinger-transformed data) together with microbial biomass and activity, according to the group of variables (**a**) (T: treatments – N source x Time; B: bacterial community; F: classified fungal community; unF: unclassified fungal community; Ac: variables of microbial biomass and active community) and (**b**) the percentage of total inertia related to each dimension.

Treatment played a major role in influencing the total data variability for bacterial and fungal community structures, and for microbial biomass and activity (Fig. 2) by including most of the data variance in the first six dimensions, ranging between 25.10% and 60.45%. Furthermore, the MFA results indicated that fungal community structure retained a relevant amount of total data variability, reaching 23.10% for the classified fungi and 17.90% for the unclassified fungi, when considering the inertia retained in the first 10 dimensions. Moreover, the microbial biomass and activity contributed 9.38% of the total data variability with values ranging between 3.15% and 19.87%. However, microbial biomass and activity only weakly influenced the total variability when compared to changes in both bacteria and fungi community structures.

The HMFA based on bacterial and fungal community structure (DNA-based) resulted in four clusters (Supplementary Fig. S6c), which increased to six clusters after adding microbial biomass and activity variables (Fig. 1c). The six clusters are related to each organic amendment; however, the Manure treatment was split into two clusters (Fig. 1C4,C5). In the Manure cluster (Fig. 1C4) are included two Straw measurements at day 69 of nutrient addition, while the other Manure cluster (Fig. 1C5) contained the remaining measurements for this treatment.

Furthermore, the v test helped to identify variables that contribute to each cluster formation (Supplementary Table S2). For cluster 1, representing the Straw treatment, we observed a strong, positive influence of fungal biomass, leucine incorporation, fungal/bacterial biomass ratio, and fungal community (genera *Columnosphaeria, Dioszegia, Rhodotorula* and two unclassified fungi from the class Hypocreomycetidea). However, most of the variables that negatively influenced this cluster corresponded to taxa related to the bacterial groups *Spartobacteria, Nitrospira* and *Acidobacteria* Gp 1 and Gp 2. Clusters 2 and 3 grouped the Maize and Lucerne treatments, respectively. Few variables contributed significantly to the formation of either cluster; active fungi and the fungal genus *Pichia* contributed to cluster 2, while the populations of the fungal genera *Otidea*, one unclassified fungi from Saccharomycetales and the bacterial population of Verrucomicrobia comprised cluster 3. Clusters 4 and 5 grouped all the Manure samples and two samples of the Straw treatment (69 days of organic addition). Cluster 4 was chiefly comprised seven different fungal genera (*Candida, Thanatephorus, Filobasidium*, and four unclassified fungi), while cluster 5 included greater abundances of six fungal genera (*Paraglomus, Stropharia, Verticillium, Glomus*, and two unknown fungi, respectively, from the family Sporodiobolales and the class Glomeromycetes) and smaller populations of an unclassified fungus from the class Pezizomycotina. Bacteria also played a different role in the formation of both clusters 4 and 5. *Chthonomonadetes* was highly significant in cluster 4, while cluster 5 included populations of *Clostridia, Bacteroidia, Alphaproteobacteria*, and *Acidobacteria* Gp 17. Finally, cluster 6 grouped all Control (inorganic N addition) samples. Eight significant variables presented here also appeared in other clusters, but with a distinct trend, especially when compared to cluster 1. Fungal biomass and Leucine incorporation presented significantly smaller values within cluster 6, and the bacterial groups *Spartobacteria, Acidobacteria* Gp 1, Gp 2 and *Nitrospira* showed greater abundances within this cluster. For cluster 6, it is also important to note the influence of the bacterial populations *Armatimonadia*, TM7 of class incertae sedis, and fungal genera *Ambispora, Hypoxylon, Verticillium, Cryptococcus*, and two fungi of unclassified genus, classified to the class-level of Sordariomycetes and phylum-level of Ascomycota, respectively.

### The role of microbial community structure and activity in the fate of nitrogen

**Figure 3:**
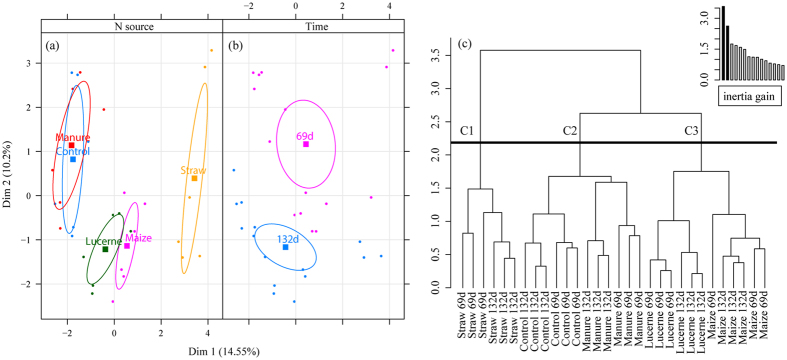
Multiple factor analysis of the bacterial and fungal community (Hellinger-transformed data) together with the microbial biomass and activity and C and N mineralization rate, according to the (**a**) organic amendment (Control, Manure, Lucerne, Corn and Straw) and (**b**) time (Nov and Jan) and (**c**) Hierarchy Clustering from the Multiple factor analysis of the bacterial and fungal community Hellinger-transformed data sets together with the microbial biomass and activity and C and N mineralization rate and the amount of inertia gained in the clustering cut-off. C1: cluster 1, C2: cluster 2, C3: cluster 3.

**Figure 4:**
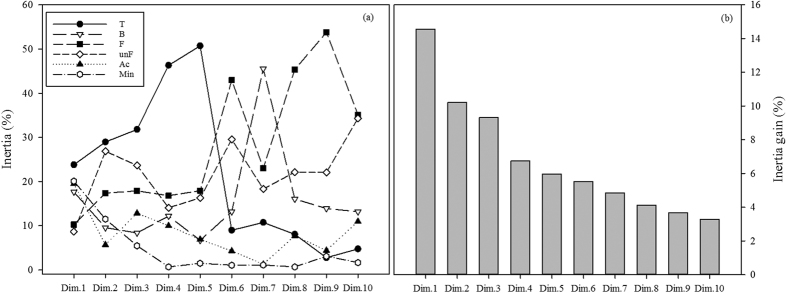
Percentage of inertia for each of the first 10 dimensions from the Multiple factor analysis of the bacterial and fungal community (Hellinger-transformed data) together with the microbial biomass and activity and C and N mineralization rate, according to the group of variables (**a**) (T: treatments – N source x Time; B: bacteria community; F: classified fungal community; unF: unclassified fungi community; Ac: variables of active community and microbial biomass; Min: carbon and nitrogen mineralization rate) and (**b**) the percentage of total inertia related to each dimension.

Straw treatment defined a distinct group, while the other treatments formed two different groups, Lucerne-Maize and Control-Manure (Fig. 3a), which became more evident after HMFA (Fig. 3c). The results again pointed to a major role of the treatment in explaining the data variability (Fig. 4a) by retaining most of the inertia in the first five dimensions, ranging between 23.51% and 50.72% of total data variability. Interestingly, the addition of variables related to nutrient mineralization reduced the variance between groups of organic amendment, as well as the differences in time (Fig. 3a,b). The grouping is more clearly observed after HMFA (Fig. 3c): Cluster 1 containing the Straw treatment, cluster 2 comprising Control and Manure treatments, and cluster 3 comprising Lucerne and Maize treatments. Table 1 presents the statistically relevant variables for the composition of each cluster. Variables that determined the Straw clustering included fungal biomass, fungal biomass to leucine incorporation ratio, bacterial biomass, carbon mineralization rate and leucine incorporation. Most of the taxa that contributed to the clustering of the Straw treatment belonged to the fungal genera *Nais, Thanatephorus*, and one fungus of unclassified genus belonging to the subclass Hypocreomycetidae, all present in high abundance. Only two groups of Bacteria were in greater abundance, the *Actinobacteria* and *Bacteriodetes* of class incertae sedis. In general, most of bacterial groups belonging to *Acidobacteria* Gp1, Gp2 and Gp4, *Nitrospira* and *Betaproteobacteria*, appeared in lower abundance within cluster 1. Potential N mineralization was also reduced within this cluster.

Cluster 2 (Manure and the Control treatments) shared a total of 10 variables with clusters 1 and 2, but the relationships differed. The strongest variables that contributed to cluster 2 were inversely related to the other treatments; these variables were the bacterial groups *Acidobacteria* Gp1, Gp2, Gp4 and two unclassified fungi (belonging to the family Hypocreales and the class Pezizomycotina). Fungal biomass, Potential C mineralization, Leucine incorporation, and bacteria belonging to *Actinobaceria, Bacteriodetes* of class incertae sedis, *Deltaproteobacteria, Verrucomicrobiae* strongly influence clusters 1 or 3, but weakly influenced cluster 2. Furthermore, five variables were statistically relevant only to cluster 2: the bacteria from the class *Spartobacteria, Acidobacteria* Gp7, *Bacilli* and the *Gammaproteobacteria*, and the fungi of unclassified genus from the family Hypocrales. Active fungi, bacterial members of *Bacilli* and *Gammaproteobacteria* and one unclassified fungus were less abundant within this cluster.

Cluster 3 (Lucerne and Maize treatments) had distinct contributing variables compared to those for cluster 1. However, two variables, Potential N mineralization and *Acidobacteria* Gp1, were common to both clusters. Cluster 3 was mainly determined by elevated potential N mineralization, *Deltaproteobacteria* and *Verrucomicrobiae* populations, and one fungal population of class *Pezizomycotina*.

According to the information contributed by each analysis type, we could detect different patterns of influence on the microbial community. The analysis of the bacterial and fungal communities based only on soil microbiome DNA revealed four different clusters and a minor role for time. Inclusion of microbial biomass and activity data improved clustering and identification of groups responding to each treatment. Inclusion of C and N mineralization rates reduced the clustering effect to three main clusters.

## Discussion

The mineral fertilizer and the four organic amendments (straw, lucerne, manure and maize) affected the microbial community due to their differences in both C/N ratio and degradability. The addition of mineral fertilizers or low degradable organic amendments with low C/N ratio (solid fraction of manure) favoured abundance of bacteria groups, while the treatments with high C/N ratio favoured abundance of fungal groups. The different chemical compositions (low to high degradability) of the applied organic amendments affected bacterial and fungal communities based on DNA, microbial biomass and activity results by inducing short-term changes. These community shifts became more pronounced when we combined the microbial structure with activity. Thus, our multifaceted approach allows a deeper understanding of short-term responses of microbial communities after the addition of organic amendments.

Changes in the microbial community resulted from a combined effect of treatment and time, and the BCA method allowed us to isolate the contribution of each effect. However, time had a minor effect on the bacterial and fungal communities, which became more evident by using MFA analyses. We also did not find any significant interaction between fungal and bacterial communities as shown by STATICO analysis. Effects of organic amendments on microbial community structure are often reported for long-term experiments ranging from 21 to 31 years^19^,^20^,^21^. Because we evaluated short-term effects of organic amendments, this may explain why we found a weak effect of time in changing the total microbial community based on DNA analysis. However, we did find that organic amendment quality was paramount to determining short-term changes in the microbial community.

It is also important to note the combined impact of exogenous microbial community together with the type of organic amendment applied. Despite that our analysis was not particularly aimed at describing the community, we know from results of previous studies that, in general, these organisms produce a primer effect^22^ which likely disappears within a few days after addition of an organic amendment^23^. Therefore, our choice of sampling periods was intended to avoid compromising the analysis of organic amendment quality in the soil microbial community. However, to determine when the effect of exogenous community ceases and the effect of nutrient addition begins remains an open question for future investigations that can make use of more intensive monitoring.

Not all microorganisms in soil are active^24^; many exist in a dormant state and prolong resumption of activity until appropriate environmental conditions are present. Therefore, dormant microbes also contribute to the microbial seedbank^15^. Thus, soil DNA sequencing reflects the microbial seedbank, while the measurements of biomass and activity relate only to the active microbial community component of the microbial seedbank. Marschner, *et al*.^21^ reported a long-term (31-year) effect of successive organic amendments on the total microbial community, evaluated by means of DGGE methodology. This suggests that, on a long-term scale, time may have an influence on the soil microbial seedbank under conditions of continuous organic amendment. In contrast, in our short-term study, time had little influence on microbial community variability under conditions of organic amendment. We propose that a successional process bridges the two time scales, where changes in the active microbial community cause changes in the soil microbial seedbank over time. Further investigation will be required to fully support this hypothesis and determine the frequency of organic amendments required to convert the changes in soil microbial activity in a strong rearrangement of soil microbial seedbank.

Positive or negative shifts in microbial abundances within treatment clusters suggests specific ecologic and metabolic interactions between bacterial and fungal soil communities. Here, the Straw cluster associated positively with leucine incorporation (bacterial activity), and the bacterial group with significant abundance was *Actinobacteria*, a known slow-growing bacteria with high metabolism^25^. This may indicate that the composition of the bacterial community shifted to a predominance of k-strategists influenced by resource availability^17^.

In our study, changes in soil nutrient resources more strongly affected the microbial biomass and activity than the abundance of various populations. The most extreme treatments - low quality organic amendment of straw versus the addition of inorganic fertilizers - changed the abundance of microbial populations, microbial biomass and activity; easily degradable organic amendments affected only a few groups of microbes. We hypothesize that the total soil microbial community may have acted as a buffer to treatments as a result of the resistance and resilience of the bacterial and fungal communities^26^. Accordingly, changes in some organic inputs promote the growth and activity of those microorganisms that are able to decompose new soil resources. In the long-term with successive organic inputs, shifts in microbial populations may affect the overall microbial seedbank depending on the community resilience and resistance^14^. Changes in microbial community structure are likely to reflect the adaptation of the microbial seedbank to new available resources, thus confirming the weak resistance of soil microbial community to disturbances. However, the amount of organic amendment applied to the soil microbial community showed resilience and changes in the microbial community over time remained a minor effect.

Moreover, as shown by the MFA results, changes in the fungal community (both classified and unclassified) explained part of the total data variability. This supports the findings of Zhang, *et al*.^1^, where the addition of straw (84.8 C/N ratio) increased the fungal biomass and altered the community structure differently compared to treatment with green manure addition (16.1/1C/N ratio) for their study of paddy soils. Thus, the quality of organic amendments selectively affects the fungal communities more than the bacterial communities. Our MFA confirm this statement by evaluating the amount of variance related to both bacterial and fungal groups, and showed that the fungal groups contributed more to the total data variability than the bacterial group. Furthermore, the cluster analysis showed that only the *Actinobacteria* population appeared in high abundance in the Straw treatment (the highest C/N ratio). Because the bacterial and fungal communities are interdependent, interacting with each other and with the ecosystem^16^, the use of MFA methodology contributes toward unraveling the ecological role of each community.

It has been well established that changes in organic matter quality impact the structure (abundance and composition) and activity of the bacterial communities^21^. However, until now it has been difficult to establish a clear relationship between changes in microbial activity and changes in the total microbial community composition. Our deep statistical analysis showed that the bacterial and fungal community structures independently showed less sensitivity than joint analysis with microbial biomass and microbial activity. This was evident by the increased number of clusters revealed by our joint analysis. However, not all changes in activity necessarily result in changes in nutrient mineralization rates as shown by the merging effect in Fig. 3c. Therefore, changes in C and N mineralization seem to reflect a net effect of both bacterial and fungal community changes, likely an outcome of functional redundancy^27^ as many of the identified microorganisms are capable of organic amendment decomposition and consequently, can mineralize nitrogen. Furthermore, the quality of the organic amendment might favour microbes that could outcompete or facilitate plant nutrient uptake. According to Boer, *et al*.^9^, microorganisms with a high-affinity uptake system may overcome the limitations of low substrate concentrations. Results of our HMFA show that fungal taxa, fungal biomass and activity determined the straw treatment clustering. This indicates that the fungal community easily colonized soil incorporated with straw because of their ability to metabolize the increased amount of C (cellulose, lignins, *etc.*) added^28^. Due to the ability of fungal hyphae to translocate nutrients^29^, fungi are able to outcompete plants in nutrient acquisition, so the addition of low quality organic amendment, in the short-term, may reduce plant nitrogen uptake.

The presence of arbuscular mycorrhizal fungi (*Paraglomus, Glomus, Ambispora* and one unclassified *Glomeromycetes*) in the clustering of manure and inorganic fertilization indicates the association of these fungi with nitrogen availability. According to Egerton-Warburton and Allen^30^, nitrogen enrichment contributes to the proliferation of small-spored species. Given the capacity of arbuscular mycorrhizal fungi to acquire P from soil, a nitrogen-rich environment may promote their development, since phosphorus becomes more limiting^31^.

Most of the statistically relevant microorganisms described here are known to be associated with soil metabolic functions. The *Nitrospira* are a group of mostly uncultured nitrite-oxidizing bacteria^32^ and we found this group to be more relevant to the inorganic fertilization treatment (Control) than to the Straw treatment. This result was probably due to the higher C/N ratio of straw, which immobilizes N, reducing its mineralization and the availability of mineral nitrogen^21^.

Furthermore, our results for the inorganic fertilizer treatment (control) showed a decreasing trend of *Acidobacteria* abundance with inorganic N addition, in contrast to results reported by Fierer, *et al*.^17^. *Acidobacteria* is a very broad and phylogenetically diverse phylum; a more detailed analysis at the subgroup level revealed that they respond differently to soil chemistry^10^,^33^. Our results pointed to the strong association between *Acidobacteria* subgroups 1, 2 and 17 with soil amended with inorganic fertilizers. A similar discrepancy was observed regarding our group of *Bacteroidetes* (an OTU of class *incertae sedis*) and a class of *Gammaproteobacteria*, which according to Fierer, *et al*.^17^ typically become increased in high N plots. These apparent inconsistencies highlight the need for a deeper characterization of microbial r/k-strategists at taxa levels.

Many organic amendments contain polymeric compounds, which depend on the microbial production of extracellular enzymes to brake them down. As the ability to degrade cellulose is widespread among fungi, especially in *Ascomycota* and *Basidiomycota*^34^, this may explain the abundance of several decomposers, such as *Candida, Columnosphaeria, Thanatephorus, Pichia, Otidea, Verticillium, Hypoxolon*^35^, *Filobasidum* and *Rhodotorula, Stropharia, Setosphaeria*^36^, *Cryptococcus* and *Dioszegia*, and also unclassified fungi belonging to *Hypocreales* and *Hypocreomicetidea, Sordariomycetes* and *Sordariomycetidae, Dothideomycetes, Tremellales, Pezizomycotina, Sporidiobololales, Trichocomaceae* and *Saccharomycetales*^37^. However, a detailed functional role for many fungal genera remains unclear.

The same problem limits detailed functional knowledge of bacterial groups. Previous studies recognized the phylum *Proteobacteria* as one of the most abundant in soil, although less than half of sequences classified as *Proteobacteria* were assigned to lower taxonomic levels^38^, especially for *Alphaproteobacteria, Betaproteobacteria, Gammaproteobacteria* and *Deltaproteobacteria*^39^. The lack of finer taxonomic information points to uncertainty when trying to relate these groups to soil functions. The same phenomenon occurs for *Clostridia, Bacteriodia, Bacilli, Chtonomonadetes, Verrucomicrobia*^40^ and *Spartobacteria*.

Not all bacteria detected in this study were assessed by the leucine incorporation method. This might be because (i) not all bacteria were active^15^ and/or (ii) bacterial groups that take up leucine may differ greatly in total bacterial abundance^39^. We must consider that leucine incorporation reflects total bacterial protein synthesis, so it represents the net metabolic activity from bacterial communities where various taxa incorporate leucine at distinctly different rates. Also, this lack of relationship may also be an outcome of the prevalence of slow-growing microorganisms with high metabolic activity.

In summary, our multifaceted approach (rRNA gene marker pyrosequencing, microbial biomass and growth, and mineralization rates) together with multivariate analyses allowed for a more thorough exploration of soil microbial dynamics following organic amendments. Although the C/N ratio and degradability differed in the four organic amendments, these treatments did not drastically change the net effect of C and N mineralization. We can therefore assume that, while the organic amendments favour the growth of microorganisms, contrasting responses of bacterial and fungal communities result in minor changes of C and N mineralization. Changes in microbial community structure likely reflect changes in total microbial biomass and activity of both bacterial and fungal communities, which shifts with new and changing resources. In the end, the appropriate use of organic amendments to increase the levels of plant nutrient uptake should take into account (i) the role of both bacterial and fungal community compositions according to their r/K-strategy, (ii) the successional process in the microbial seedbank composition and (iii) the net effect of C and N mineralization.

## Material and Methods

### Soil and organic material

A pot study was carried out in Wageningen, the Netherlands, from September 6, 2011 to January 16, 2012. It consisted of six treatments and a total of 54 pots with various combinations of inorganic nitrogen fertilizer and organic products (Table 2 and Supplementary Table S1). The sandy soil was characterized as having pH 6.4, C content of 20 g/kg and total N content of 0.9 g/kg. Four organic products with contrasting values of C/N ratio and degradability were mixed through the entire soil mass: the solid fraction of cattle manure (Manure; low C/N, low degradability), wheat straw (Straw; high C/N, low degradability), silage of alfalfa (Lucerne; low C/N, high degradability), and maize silage (Maize; high C/N, high degradability). Ammonium sulphate was added as inorganic N fertilizer, at concentrations of 0.98 (DControl) and 1.96 (Control) g/pot, corresponding to 200 and 400 kg N/ha, respectively. The study was carried out for 132 days, with measurements carried out at 32, 69 and 132 days.

### Microbial biomass and activity and mineralization rate

Bacterial biomass was measured by confocal laser scanning microscopy and automatic image analysis^41^. Fungal biomass and the percentage of active hyphae were determined by epifluorescence microscopy after staining of soil smears with Differential Fluorescent Stain (DFS)^42^. DFS stains active hyphae red because of a higher content of nucleic acids. Bacterial growth rate was determined by incorporation of ^14^C-leucine into proteins during a short (1 h) incubation^43^. Potential C and N mineralization rates were measured by incubation of soil samples for 6 weeks at 20 °C^44^. Results of the first week were not used as these reflected initial disturbances of transfer to the microcosm. Oxygen and CO_2_ were measured weekly using a gas chromatograph (Thermo Fisher Scientific Inc., Waltham, MA, USA). Nitrogen mineralization rates were calculated from the increase in mineral N (nitrate and ammonium) between week 2 and week 6, which served as a proxy for net mineralization of plant-available N. We evaluated the mineralization rates for only the later two time points (69 d and 132 d).

### Soil DNA isolation, PCR and pyrosequencing

Aliquots (0.3 g) of each pot were taken for total community DNA extraction. The Power Soil kit (MolBio, Carlsbad, CA) protocol was followed with the modification of 5.5 m·s^−1^ for 10 min bead beating. Total DNA concentrations were measured using a ND-1000 spectrophotometer (Nanodrop, Wilmington, DE).

### 16S and 18S rRNA genes amplicon library preparation

The 16S rRNA gene marker was amplified from soil DNA of each sample. Amplicons for barcoded pyrosequencing were obtained using the forward primer 515 F (5′-GTGCCAGCMGCCGCGGTAA-3′) and the reverse primer 806 R (5′-GGACTACVSGGGTATCTAAT-3′) for bacteria and the primers FR1 (5′-AICCATTCAATCGGTAIT-3′) and FF390.1 (5′-CGWTAACGAACGAGACCT-3′) for fungi based on the methods described in Verbruggen, *et al*.^45^. The 515 F primer included the Roche 454-A adapter, a 10 bp barcode and a GT linker, and the reverse primer included the Roche 454-B adapter, the same 10 bp barcode as the 515F primer, and a GG linker. For the 18S rRNA gene markers the Roche MID tag IDs 24 to 26 and 61 to 69 were added to barcode the samples. Amplification reactions were performed using 5 μM of each forward and reverse primer, 5 mM dNTPs (Invitrogen, Carlsbad, CA), 0.5 μL of BSA (4 mg/mL), 1 unit of Taq polymerase (Roche, Indianapolis, IN), and 1 μL of sample DNA as the template in a total volume of 25 μL. The PCR was conducted with an initial incubation of 5 min at 94 °C, followed by 25 cycles of 1 min at 94 °C, 1 min at the annealing temperature of 53 °C for bacteria and 57 °C for fungi amplification and 1 min at 72 °C, followed by a final incubation of 10 min at 72 °C. Each sample was amplified in three reactions, and resulting amplification products were pooled to achieve equal mass concentrations in the final mixture.

### Data analyses

The 16S rRNA partial gene sequence data was processed using the QIIME pipeline on a local installation of Galaxy^46^,^47^. Low-quality sequences (less than 150 bp in length or average quality score smaller than 25) were removed. Denoising and chimera checking were performed using USEARCH and UCHIME^48^,^49^. The Operational Taxonomic Units (OTUs) were identified using USEARCH with a phylotype defined at the 97% sequence similarity level. For the 16S rRNA data, representative phylotype sequences were aligned in Galaxy using PyNAST against the GreenGenes Ribosomal Database (available at http://greengenes.lbl.gov). The taxonomic ranks were annotated in place of the six GreenGenes taxonomic ranks.

For the 18S rRNA gene data, representative phylotype sequences were aligned using PyNAST against the SILVA RDP Database^50^. The taxonomic groups were specified in the analysis in place of the fifteen taxonomic ranks from the SILVA annotation.

### Statistical analysis

All statistical analyses were performed in R v3.2.3^51^. The sequenced datasets (bacterial and fungal) were processed using the “phyloseq” package in R^52^. The bacterial and fungal OTU abundances were summarized at the Class and Genus taxonomical levels, respectively, and the Hellinger transformation was adopted prior to ordination methods^53^ in order to stabilize the mean-variance relationship and avoid to confound location and dispersion effects^54^.

The “ade4” R package^55^ was used to compare the effect of treatment on bacterial and fungal community structures (DNA only). Between-class analysis (BCA) explored dissimilarities between the treatments and the time for each community (bacteria and fungi) by measuring the amount of variance restricted to the grouping factor as a percentage of the total inertia^55^,^56^. BCA is an alternative method to linear discriminant analysis where the number of samples can be smaller than the number of variables^57^. Principal Component Analysis (PCA) was applied to each community dataset prior to the BCA. A Monte-Carlo test of the treatment groups was performed with 9999 permutations.

After BCA analysis, we arranged the dataset into two k-tables (sampling pot x community x time) for the bacterial and fungal communities, respectively^58^. The common structure between both k-tables were assessed by STATICO and COSTATIS methods, by generating a compromise table (a table of covariances, which we called “co-structure”) where each method gives a different emphasis for the time effect^58^. STATICO method provided information about the overall co-structure in time, while COSTATIS evaluates the relationships between the two stable structures (the compromise tables of both bacterial and fungal communities structure) by maximizing the co-inertia for the various treatments.

Subsequently, the joint effect of variable groups was evaluated by Multiple Factor Analysis (MFA) using the R package “FactoMineR”^59^. MFA is a variant of co-inertia analysis that seeks the common structure present for various variables groups^60^. This method consists of a two-step principal component analysis that balances the inertia between different groups of variables. Moreover, it is possible to determine the amount of data variability related to each subset. For this analysis the following groups of variables were selected: bacterial community, fungal community (divided into classified and unclassified groups), and the standardized variables of microbial biomass and activity, and nitrogen and carbon mineralization rates.

To evaluate how each variable is hierarchically structured^60^, we performed a Hierarchical Clustering of a Multiple Factor Analysis (HMFA). The clustering cutoff criterion was the amount of inertia that retained most of data variability. We evaluated which variables contributed more to each cluster through the v test (a test value)^60^ and the *p*-value was adjusted to control for the false discovery rate (q < 0.05)^61^. The v test measures the standardized deviation of each selected category (in our case, the clusters) and reports the probability of having an extreme value (whether positive or negative) than the overall mean of the general average. This analysis enabled us to investigate which variables contribute to characterize each of the clusters and identify if this occurs because of a value smaller (negative value) or larger (positive value) than the overall mean value.

## Additional Information

**How to cite this article**: Leite, M. F. A. *et al*. Organic nitrogen rearranges both structure and activity of the soil-borne microbial seedbank. *Sci. Rep.* **7**, 42634; doi: 10.1038/srep42634 (2017).

**Publisher's note:** Springer Nature remains neutral with regard to jurisdictional claims in published maps and institutional affiliations.

## Supplementary Material

Supplementary Material

## Figures and Tables

**Table 1:** Variables relevant to the hierarchical clustering from the multiple factor analysis of the bacterial and fungal community Hellinger-transformed data sets together with the microbial biomass and activity and C and N mineralization rates.

Variables	V test value		
	Cluster 1 (Straw)	Cluster 2 (Manure and Control)	Cluster 3 (Lucerne and Maize)
Fungal biomass (μg C/g dry soil)	4.75	−2.9	
Potential C mineralization (mg C/kg.week)	4.57	−3.57	
Potential C mineralization based on O_2_ consumption (mg C/kg.wk)	4.56	−3.70	
Fungi biomass/Leucine incorporation	4.48		
Fungi/Bacteria (C/C)	4.17		
Unclassified (Hypocreomycetidea)	3.48		
Bacteroidetes *incertae sedis* class *incertae sedis*	2.98	−2.90	
*Nais*	2.87		
Leucine incorporation (pmol/g.h)	2.78	−3.53	
Actinobacteria	2.44	−3.76	
*Thanatephorus*	2.44		
Acidobacteria Gp4	−2.44	3.52	
Acidobacteria Gp1	−2.53	4.56	−2.49
Nitrospira	−2.76		
Acidobacteria Gp2	−2.77	3.00	
N mineralization/mineralizable N	−3.35		
Betaproteobacteria	−3.61		
Potential N mineralization (mg N/kg.wk)	−3.90		2.83
Unclassified (Pezizomycotina)		−2.88	3.47
Deltaproteobacteria		−3.13	2.68
Verrucomicrobiae		−2.06	2.59
Unclassified (Sordariomycetes)			−2.57
Spartobacteria		3.61	
Acidobacteria Gp7		3.45	
Bacilli		−2.77	
Unclassified (Hypocreales)		−2.86	
Gammaproteobacteria		−2.97	

**Table 2:** Sources of N applied in the study.

Treatment	Fertiliser N (g N /pot)	Organic product	C/N ratio	Organic product (g/pot)	N in organic product (g N/pot)	Total N input (g N/pot)
DControl	0.98	none	—	0	—	0.98
Control	1.96	none	—	0	—	1.96
Manure	0.98	Cattle manure	19.1	193	0.98	1.96
Straw	0.98	Wheat straw	133.4	278	0.98	1.96
Lucerne	0.98	Alfalfa silage	11.8	113	0.98	1.96
Maize	0.98	Maize silage	42.9	216	0.98	1.96
