# Supplementary material for: Organic nitrogen rearranges both structure and activity of the soil-borne microbial seedbank
Source: Sci Rep. 2017 Feb 15;7:42634. doi: 10.1038/srep42634 (PMC5309777; doi:10.1038/srep42634)
Supplement: Supplementary Material [file srep42634-s1.pdf]

Organic nitrogen rearranges both structure and activity of the soil-borne microbial seedbank

Márcio F. A. Leite[1,2], Yao Pan[1], Jaap Bloem[3], Hein ten Berge[4], Eiko E. Kuramae[1*]

[1]Netherlands Institute of Ecology (NIOO/KNAW), Department of Microbial Ecology, Wageningen

[2]Agroecology Program of Maranhão State University – UEMA, São Luís – MA, Brazil.

[3]Alterra, Wageningen UR

[4]Plant Research International, Wageningen UR

Supplementary Material

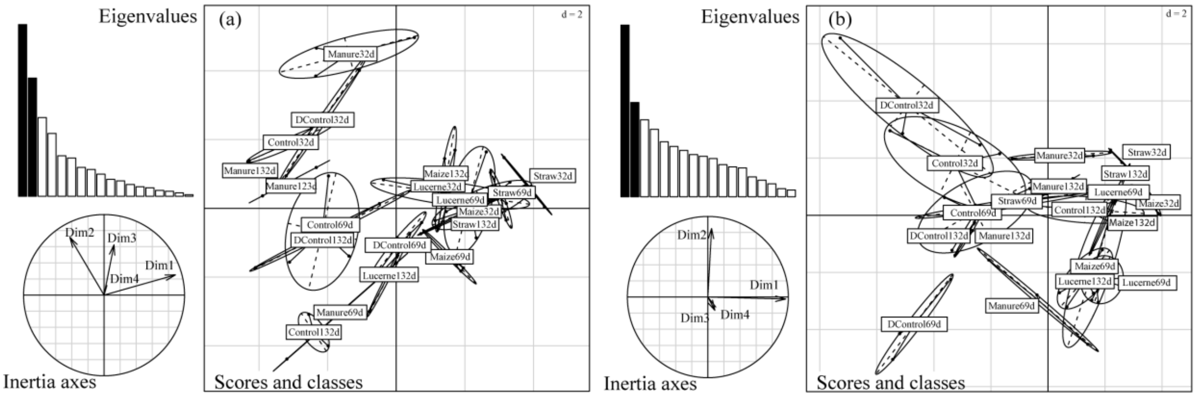

Figure S1. Between-Class Analysis for (a) bacterial and (b) fungal community structures according to the measured time (32, 69 and 132 days) and organic amendment: Control, DControl, Manure, Lucerne, Maize and Straw. Correspondence analysis (CA) was applied to the community samples. The inertia between class was 56.03% for bacteria and 44.25%, for fungi and the Monte Carlo permutation level of significance was p=0.001 for bacterial and p=0.001 for fungi.

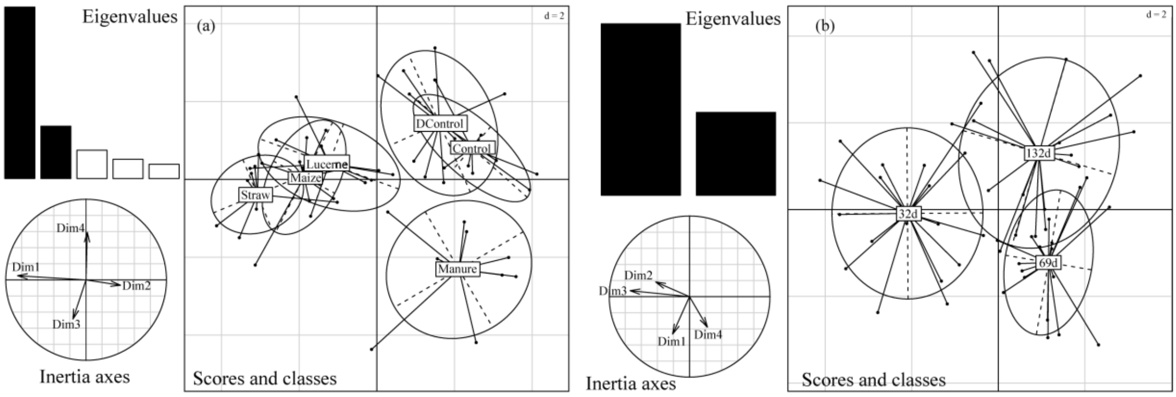

Figure S2. Between-Class Analysis of bacterial communities according to (a) organic amendment (Control, DControl, Manure, Lucerne, Maize and Straw), and (b) time (32, 69 and 132 days).

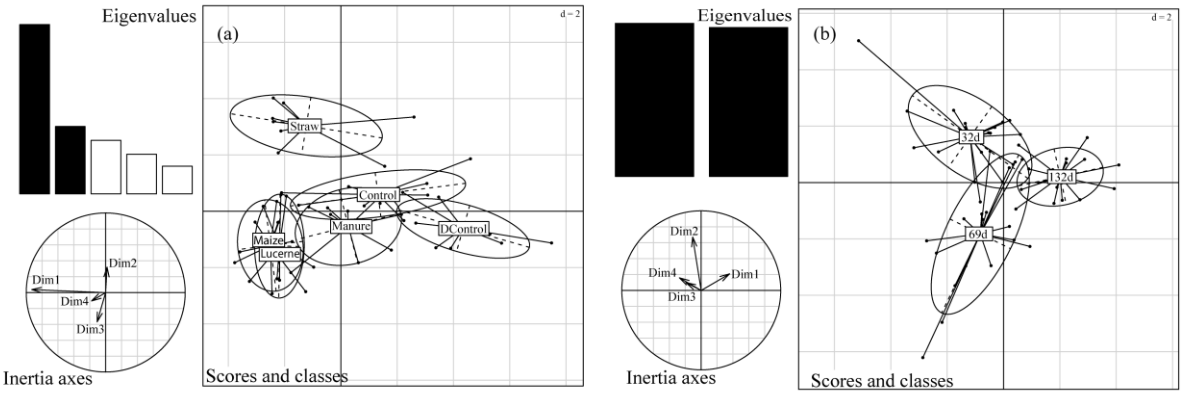

Figure S3. Between-class analysis of the fungal community according to (a) organic amendment type (Control, DControl, Manure, Lucerne, Maize and Straw) and, (b) time (32, 69 and 132 days).

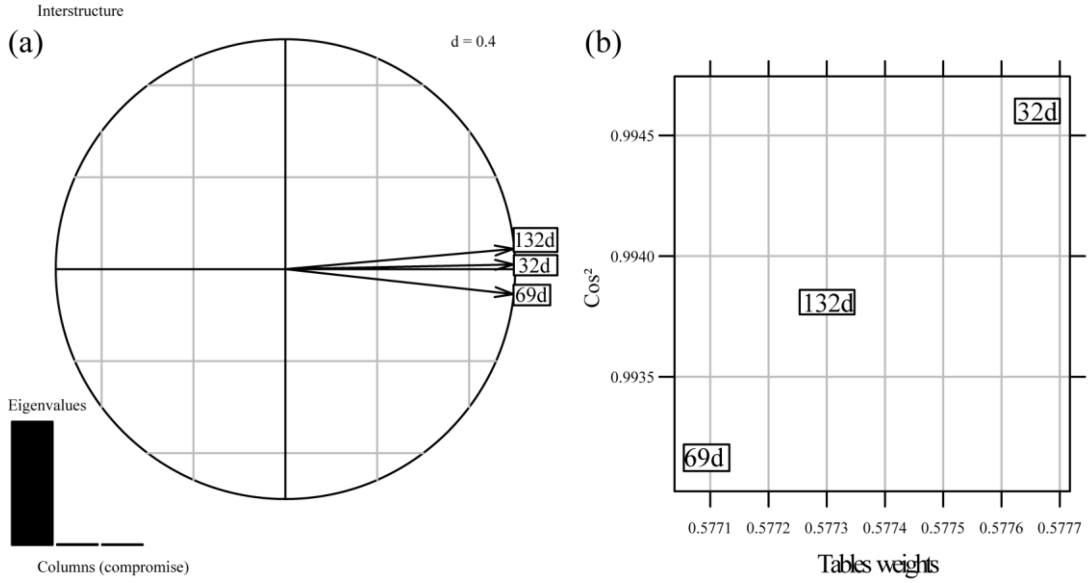

Figure S4. STATICO analysis of both bacterial and fungal communities according to time (32, 69 and 132 days) and organic amendment (Control, DControl, Manure, Lucerne, Maize and Straw). Correspondence analysis (CA) was applied to the community samples. The scale is given by the value "d" in the upper right corner of the compromise plot and corresponds to the size of the background grid. The interstructure plot (a), showing the three sampling periods, and the importance of the corresponding tables in the definition of the compromise (coordinate of the points on the first axis). Typological values (b) for the three tables (square cosines vs. table weights).

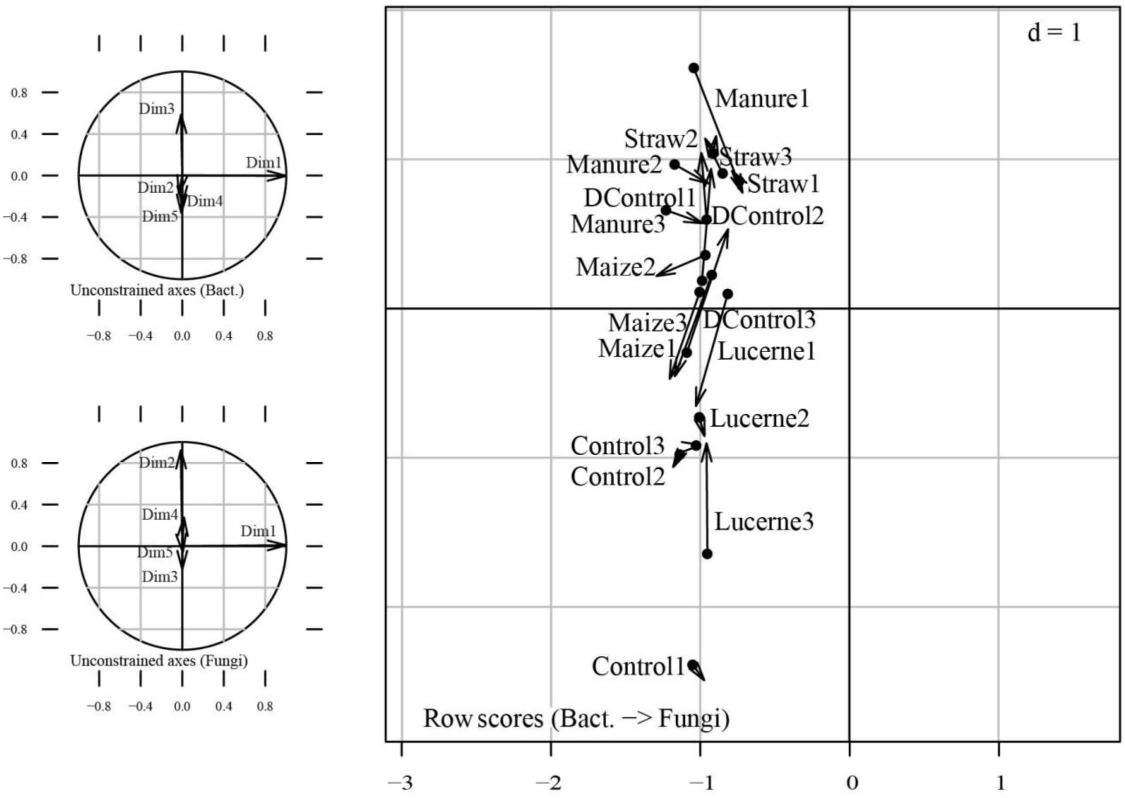

Figure S5. COSTATIS analysis for both bacterial and fungal communities according to time (32, 69 and 132 days) and organic amendment (Control, DControl, Manure, Lucerne, Maize and Straw).

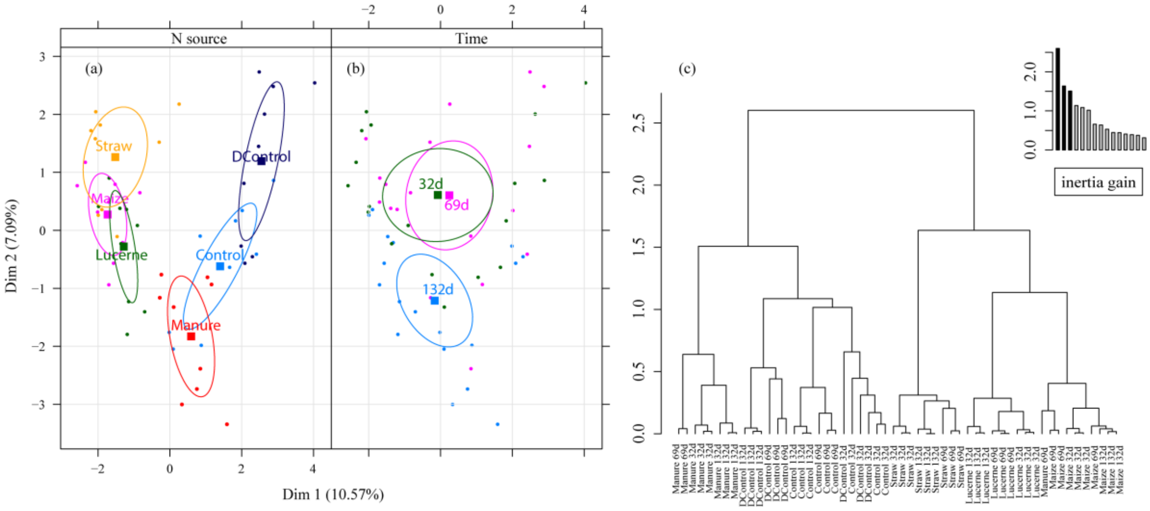

Figure S6. Multiple factor analysis of the bacterial and fungal community data sets according to (a) organic amendment (Control, DControl, Slurry, Lucerne, Corn and Straw), (b) time (32, 69 and 132 days), and (c) Hierarchy of Multiple factor analysis considering the groups of variables corresponding to the bacterial and fungal communities (Hellinger-transformed data), to the organic amendment (Control, Manure, Lucerne, Maize and Straw) and to time (32, 69 and 132 days) and the amount of inertia gained in the clustering cut-off.

Table S1- Digestibility of organic inputs

	NDF	ADF	ADL	Sugars, proteins, fats, etc	Starch	Hemi-cellulose	Cellulose	Lignin
	(g per kg DM)							
Cattle Manure	714	553	227	28.6%	0.0%	16.1%	32.7%	22.7%
Wheat Straw	850	515	174	15.0%	0.0%	33.5%	34.1%	17.4%
Lucerne	399	330	91	59.6%	0.5%	6.8%	23.9%	9.1%
Maize	436	246	27	17.1%	39.3%	19.0%	22.0%	2.7%

NDF: neutral detergent fiber; ADF: acid detergent fiber; ADL: acid detergent lignin. All contents on dry matter basis

Table S2- Variables relevant to the hierarchical clustering from the multiple factor analysis of of the bacterial and fungal communities (Hellinger-transformed data) together with the microbial biomass and activity.

Variables	V test value					
	Cluster 1 (Straw)	Cluster 2 (Maize)	Cluster 3 (Lucerne)	Cluster 4 (Manure 69d + Straw 69d)	Cluster 5 (Manure)	Cluster 6 (Control)
Fungal biomass (µg C/g dry soil)	4.98					-2.48
Leucine incorporation (pmol/g.h)	3.98					-2.92
Fungal biomass/ leucine incorporatio	3.83					
Columnosphaeria	3.82					
Unclassified (Hypocreomycetidea)	3.65					
Dioszegia	3.19					
Setosphaeria	2.95					
Fungi/Bacteria (C/C)	2.91					
Rhodotorula	2.74					
Unclassified (Hypocreales)	2.67					
Actinobacteria	2.48				-3.05	
Spartobacteria	-2.43					2.96
Acidobacteria Gp1	-2.46					3.65
Acidobacteria Gp2	-2.61				2.85	2.78
Nitrospira	-2.78					2.33
Acidobacteria Gp4	-2.97					
Active fungi (% of hyphal length)		3.95				
Pichia		2.99				
Otidea			2.71			
Unclassified (Saccharomycetales)			2.53			
Verrucomicrobiae			2.43			-3.02
Unclassified (Dothideomycetes)				4.26		
Candida				3.61		
Unclassified (Trichomaceae)				3.44		
Unclassified (Basidiomycota)				3.21		
Chthonomonadetes				3.07		
Thanatephorus				3.05		
Unclassified (mitosporic Tremellales)				2.84		
Filobasidium				2.84		
Clostridia					4.94	

Bacteroidia	4.56	
Paraglomus	4.2	
Stropharia	3.73	
Unclassified (Sordariomycetidae)	3.17	
Alphaproteobacteria	3.11	
unclassified (Sporodiobolales)	3.03	
Acidobacteria Gp17	2.83	
Verticillium	2.82	3.04
unclassified (Glomeromycetes)	2.57	
Glomus	2.55	
Unclassified (Pezizomycotina)	-2.55	
Unclassified (Sordariomycetes)		3.48
Hypoxylon		3.09
Armatimonadia		2.93
Unclassified (Ascomycota)		2.84
Cryptococcus		2.58
Ambispora		2.53
TM7 class incertae sedis		2.44
Gammaproteobacteria		-2.59
Bacteroidetes incertae sedis class incertae sedis		-2.68
